# Iron Uptake Mediated by Binding of H-Ferritin to the TIM-2 Receptor in Mouse Cells

**DOI:** 10.1371/journal.pone.0023800

**Published:** 2011-08-19

**Authors:** Jian Han, William E. Seaman, Xiumin Di, Wei Wang, Mark Willingham, Frank M. Torti, Suzy V. Torti

**Affiliations:** 1 Department of Biology, North Carolina Agricultural and Technical State University, Greensboro, North Carolina, United States of America; 2 Department of Medicine, University of California San Francisco, California, United States of America; 3 Veterans Affairs Medical Center, San Francisco, California, United States of America; 4 Department of Cancer Biology, Wake Forest School of Medicine, Winston-Salem, North Carolina, United States of America; 5 Department of Pathology, Wake Forest School of Medicine, Winston-Salem, North Carolina, United States of America; 6 Comprehensive Cancer Center, Wake Forest School of Medicine, Winston-Salem, North Carolina, United States of America; 7 Department of Biochemistry, Wake Forest School of Medicine, Winston-Salem, North Carolina, United States of America; National Institute of Health, United States of America

## Abstract

Ferritin binds specifically and saturably to a variety of cell types, and recently several ferritin receptors have been cloned. TIM-2 is a specific receptor for H ferritin (HFt) in the mouse. TIM-2 is a member of the T cell immunoglobulin and mucin domain containing (TIM) protein family and plays an important role in immunity. The expression of TIM-2 outside of the immune system indicates that this receptor may have broader roles. We tested whether ferritin binding to TIM-2 can serve as an iron delivery mechanism. TIM-2 was transfected into normal (TCMK-1) mouse kidney cells, where it was appropriately expressed on the cell surface. HFt was labeled with ^55^Fe and ^55^Fe-HFt was incubated with TIM-2 positive cells or controls. ^55^Fe-HFt uptake was observed only in TIM-2 positive cells. HFt uptake was also seen in A20 B cells, which express endogenous TIM-2. TIM-2 levels were not increased by iron chelation. Uptake of ^55^Fe-HFt was specific and temperature-dependent. HFt taken up by TIM-2 positive cells transited through the endosome and eventually entered a lysosomal compartment, distinguishing the HFt pathway from that of transferrin, the classical vehicle for cellular iron delivery. Iron delivered following binding of HFt to TIM-2 entered the cytosol and became metabolically available, resulting in increased levels of endogenous intracellular ferritin. We conclude that TIM-2 can function as an iron uptake pathway.

## Introduction

Ferritin is a ubiquitously distributed protein principally known for its role in iron storage and detoxification [1], [2]. It is composed of two subunit types, termed H and L; twenty-four of these assemble to form the ferritin protein. The ratio of subunits within the assembled protein is dictated by tissue type and is also modulated by exogenous stimuli [1]. The H subunit of ferritin possesses ferroxidase activity [3], and enables the oxidation of iron, whereas the L subunit facilitates iron nucleation within the central core. Up to 4500 atoms of iron can be stored in a non-toxic but bioavailable form within the central cavity of the ferritin protein.

Ferritin is regulated post-transcriptionally by iron through the action of iron regulatory proteins (IRPs), which act as ferritin translational repressors (see [4], [5] for review). When levels of intracellular iron rise, IRPs are inactivated, and ferritin mRNA is translated. IRPs also control the levels of transferrin receptor 1 (TfR1), a protein that mediates uptake of iron bound to transferrin, the principal source of iron in mammalian cells. In the case of TfR1, regulation by IRPs destabilizes TfR1 mRNA under conditions of iron repletion. Conversely, TfR1 mRNA and protein increase under conditions of iron depletion, such as that induced by an iron chelator.

Ferritin exists in multiple compartments and exhibits functions in addition to its classic role in intracellular iron storage. Ferritin is present in the cytosol of most cells, as well as in the nucleus or mitochondria of some cell types. In addition, ferritin is present in the bloodstream of mammals, and its levels can increase dramatically in inflammation and cancer [6], [7], [8]. The role of extracellular ferritin remains uncertain. Exogenous ferritin exerts immunosuppressive effects, inhibiting lymphocyte function [9], [10], [11] and affecting chemokine receptor-mediated signal transduction [12]. Ferritin also acts as a pro-inflammatory signaling molecule in hepatic stellate cells [13] and inhibits the anti-angiogenic effects of kininogen [14]. Exogenous ferritin also can serve as an iron delivery vehicle. For example, ferritin released by Kupffer cells is efficiently taken up by hepatocytes [15], and ferritin secreted by macrophages serves as an iron source for erythroid precursor cells [16].

Ferritin binds specifically and saturably to a variety of cell types [10], [17], [18], [19], [20], [21], and several ferritin receptors have recently been cloned. Mouse TIM-2 was the first ferritin receptor to be cloned [22]. It is a specific receptor for HFt (i.e. ferritin comprised of the H subunit), and is present in germinal center B cells, liver bile duct epithelial cells, and kidney renal distal tubule cells, as well as mouse oligodendrocytes [23]. Since oligodendrocytes do not express transferrin receptor (which is responsible for iron uptake in most mammalian cells), TIM-2 was postulated to be the primary mechanism for iron uptake by these cells [23]. Another cell surface receptor for ferritin is Scara5, a mouse scavenger receptor [24]. In contrast to TIM-2, which is a HFt receptor, Scara5 preferentially binds LFt (ferritin rich in the L subunit). Scara5 plays an important role in kidney organogenesis, and serves as an iron delivery vehicle to kidney capsular cells during development [24]. The only ferritin receptor identified in human cells to date is transferrin receptor 1, which binds both HFt and transferrin [25].

The consequences of ferritin binding to its receptor TIM-2 have not been assessed. TIM-2 is a member of the T cell immunoglobulin and mucin domain containing (TIM) protein family [26] and it plays an important role in immunity [27]. TIM-2 deficient mice have a heightened Th2 immune response, and they demonstrate increased lung inflammation following allergic challenge [28]. Ectopic expression of TIM-2 also impairs induction of NFAT and AP-1 in T cells *in vitro* [29]. However the expression of TIM-2 outside the immune system indicates that this receptor may have broader roles. For example, TIM-2 regulates the differentiation of mouse fetal hepatocytes [30].

In this study, we tested whether ferritin binding to TIM-2 can serve as an iron delivery mechanism in cells outside the brain. We observed that iron-loaded HFt is taken up by TIM-2 positive cells via an endocytic pathway, with delivery to lysosomes. Iron delivered via this pathway becomes metabolically available, resulting in increased levels of intracellular ferritin. We conclude that ferritin can deliver iron to cells expressing TIM-2.

## Materials and Methods

### Chemicals and cell cultures

Ferrous ammonium sulfate, nitrilotriacetic acid (NTA), ferric ammonium sulfate, phenylarsine oxide (PAO), and mouse apo-transferrin were purchased from Sigma (St Louis, MO). ^55^FeCl_3_ was purchased from Perkin Elmer (Fremont, CA). The TCMK-1 mouse kidney epithelial cell line and the A20 mouse B cell line were obtained from the American Type Culture Collection (ATCC, Rockville, MD). TCMK-1 cells were maintained in PC-1 serum-free complete medium purchased from Lonza (Allendale, NJ) supplemented with 100 units/ml penicillin, 100 µg/ml streptomycin, and 2 mM glutamine. A20 cells were maintained in RPMI 1640 medium from Invitrogen (Carlsbad, CA) supplemented with 2 mM L-glutamine, 1.5 g/L sodium bicarbonate, 4.5 g/L glucose, 10 mM HEPES, 1.0 mM sodium pyruvate, 0.05 mM 2-mercaptoethanol, and 10% fetal bovine serum (Invitrogen [Carlsbad, CA]). Cells were incubated in a humidified atmosphere containing 5% CO_2_ at 37°C.

### Establishment of TIM-2 stable clones

TCMK-1 cells from the ATCC were transfected with TIM-2 containing and empty vector plasmids (BSR-α-FLAG [22]). After 24 hours, 1000 µg/ml G418 was added to the medium for clonal selection. After selection, stable clones were maintained in medium containing 350 µg/ml G418.

### Flow Cytometry

One million A20 cells were harvested and washed with PBS. The cells were blocked with inactivated human AB serum (Innovative Research [Novi, Michigan]) for 30 min and then incubated with 1–2 µg biotin-linked anti-TIM-2 monoclonal antibody [22] in 1% BSA (Sigma Aldrich [St. Louis, Missouri])/PBS for 1 hour at 4°C in the absence of permeabilizing agents. After incubation, the cells were washed with PBS and then incubated with allophycocyanin (APC) linked streptavidin for 1 hour. The cells were washed and analyzed on a FACS Aria flow cytometer (Becton Dickinson) using Diva and DakoCyotmation Summit 4.3 analysis software.

### Immunoprecipitation

Cells were lysed in lysis buffer (0.12% Triton X-100, 0.15 M NaCl, 2.0 mM triethanolamine, pH 7.8, 2.5 µM CaCl_2_, 1 µM MgSO_4_, 0.01% sodium azide) containing freshly added 1 µg/ml aprotinin, 0.5 µg/ml leupeptin, 1 mM PMSF and 1 mM vanndate. Lysates were incubated 2–3 h on ice and clarified by centrifugation at 16,000 x *g* for 20 min at 4°C. Sixty µg protein were used for immunoprecipiatation and 15 µg for western blot. For immunoprecipitation, protein extracts were incubated with anti-TIM-2 monoclonal antibody [22] and protein G beads [Invitrogen, CA] at 4°C overnight. Beads were then washed three times with FROP buffer (150 mM NaCl, 5 mM CHAPS, 50 mM Tris-HCl, pH 8.0), suspended in 2xSDS gel loading buffer, heat denatured and resolved on a 12% SDS-PAGE gel. The gel was transferred onto a nitrocellulose membrane and blotted for TIM-2 protein using rabbit polyclonal anti-TIM-2 antibody [22] followed by HRP conjugated anti-rabbit IgG and incubation with chemiluminescent substrate (SuperSignal West Pico, Thermo Scientific). Pre-stained standards (Full-range rainbow molecular weight markers, Cat# RPN800E, GE Healthcare Life Sciences [Piscataway, NJ]) were used to estimate molecular weights.

### Recombinant H Ferritin purification and iron loading

Mouse HFt was produced in *E. coli* and purified as described [31]. Endotoxin was removed by adsorption to immobilized polymyxin B (Detoxi-GelTM, pierce Chemical Co.), according to the methods of the manufacturer. Endotoxin removal was confirmed using the limulus amebocyte lysate assay (QCL-1000, Cambrex Bio Science). To load iron into ferritin, 450 µM ferrous ammonium sulfate was added to 50 µg/ml H ferritin diluted in 0.1 M HEPES pH 6.0 [32]. Iron uptake was monitored spectrophotometrically at 310 nm. To label mouse recombinant HFt with ^55^Fe, 125 µCi ^55^FeCl_3_ was added to 50 µg/ml HFt in buffer containing 20 µM citric acid, 2 mM ascorbate, and 0.1 M HEPES (pH 6.0). This was followed by the addition of 450 µM unlabeled ferrous ammonium sulfate and incubation for an additional hour. The sample was then dialyzed in 0.1 M HEPES at 4°C overnight [33]. The final ratio of iron:ferritin was approximately 2000∶1 as measured by ferrozine assay [34]. Specific activity of the ^55^Fe-ferritin varied among preparations, and direct comparisons were always made using the same batch of ^55^Fe-ferritin. Ferritin was filtered through a 0.45 µm syringe filter prior to cell culture use.

### Real time RT-PCR

Real-time PCR was carried out on an ABI Prism 7000 sequence detection system (Applied Biosystems, Foster City, CA) as described [35]. The standard curve method was used for quantification. Total RNA was isolated using Trizol reagent according to the manufacturer's instructions. 30 µg RNA was treated with 35U DNase I (Promega) for 30 min at 37°C. After DNase I digestion, RNA was purified using Absolutely RNA RT-PCR Miniprep kit (Stratagene) following the manufacturer's protocol. An oligodT primer was used in cDNA synthesis. β-actin was measured as an internal control. Primers for PCR were designed with IDT PrimerQuest software (Intergrated DNA Technologies, Inc.). The forward primer for mouse TIM-2 was: 5′-CCAACACCAGCACACACAGAGACCT-3′, and the reverse primer was: 5′-TGGCTTCTGTGGAGGGATTACTTCA-3′.

### Electron microscopy observation

A total of 3×10^6^ TIM-2 or vector transfectants were plated on 100 mm cell culture dishes and allowed to attach overnight before 150 µg HFt (fully saturated with iron) was added. The cells were fixed in 2.5% gluteraldehyde and 2% osmium, dehydrated in ethanol, scraped, and placed into a microcentrifuge tube. The samples were infused with resin, cut into 80 nm sections, stained with 33% lead citrate, and viewed with a Philips 400 transmission electron microscope (Eindhoven, the Netherlands) at 60Kv.

### Biotinylation of ferritin and capture of ferritin using streptavidin beads

250 µg HFt was labeled with biotin using EZ-Link Sulfo-NHS-LC-Biotin kit (Pierce Chemical Co., IL) following the protocol provided by the manufacturer. Biotinylated HFt was dialyzed in PBS overnight at 4°C. 125 µCi ^55^Fe was loaded into biotinylated HFt as described above, and biotinylated-^55^Fe-HFt was then dialyzed in 0.1 M HEPES overnight. Three million TIM-2 or vector transfectants were plated in 100 mm cell culture dishes and allowed to attach overnight. Twoµg/ml biotinylated-^55^Fe-HFt (approximately 4 nM) was added to each plate and incubated at 37°C for 2 hours. Plates were then washed 3 times with PBS and placed in growth media. Cells and media were collected at 0, 2, 4, 8, 24, and 48 h after incubation with biotinylated-^55^Fe-HFt. Cell lysates were prepared by homogenization in whole cell lysis buffer (25 mM Tris pH 7.4, 1% Triton X-100, 1%SDS , 1% sodium deoxycholate, 150 mM NaCl , 2 µg/ml aprotinin, 1 mM PMSF, complete protease inhibitor [Roche]) for 5 seconds. Samples were clarified by centrifugation at 12,000 x *g* at 4°C for 15 minutes. Biotin-^55^Fe-HFt was immunoprecipitated from the supernatant by incubation with streptavidin conjugated beads (Streptavidin –APC [Jackson Immuno Research, PA]), followed by western blot detection for HFt using polyclonal anti-HFt antibody [35]. Extracts depleted of biotinylated ferritin by incubation with streptavidin beads were used for western blot detection of endogenous HFt.

## Results

### TIM-2 mediates uptake of ferritin-bound iron

**Figure 1:**
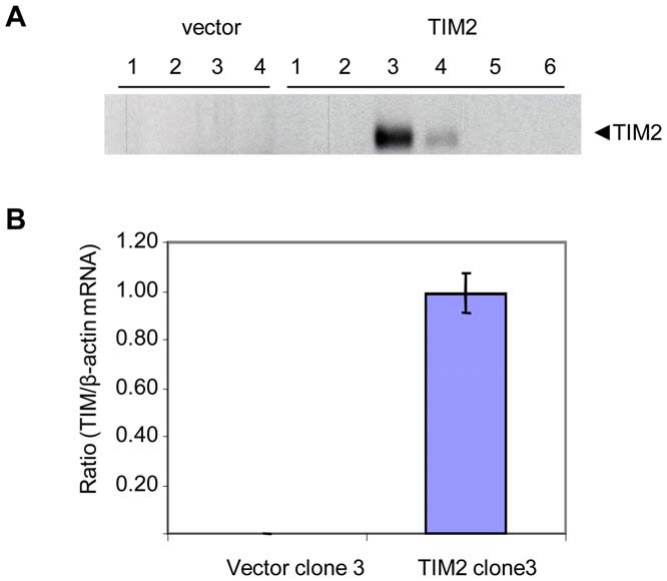
Transfected TCMK-1 cells express TIM-2. A: Stable transfectants were examined for TIM-2 protein expression by immunoprecipitation followed by western blot. B: A TIM-2 positive clone (#3) and a control vector transfectant (#3) were tested for TIM-2 mRNA expression by realtime RT-PCR (means and standard deviation, n = 4).

To test whether TIM-2 could function in iron uptake, we used a mouse kidney epithelial cell line, TCMK-1. Since these cells do not express detectable endogenous TIM-2, they provided a clean background in which to specifically assess the contribution of TIM-2 to ferritin-dependent iron uptake. TCMK cells were transfected with a TIM-2 expression vector or an empty vector control, and stable transfectants were selected. TIM-2 expression in the transfectants was examined at both protein and mRNA levels (Figure 1). As expected, some TIM-2 transfectants expressed TIM-2, but no TIM-2 was discernable in vector controls as measured by western blot (Figure 1A) or real time RT-PCR (Figure 1B).

**Figure 2:**
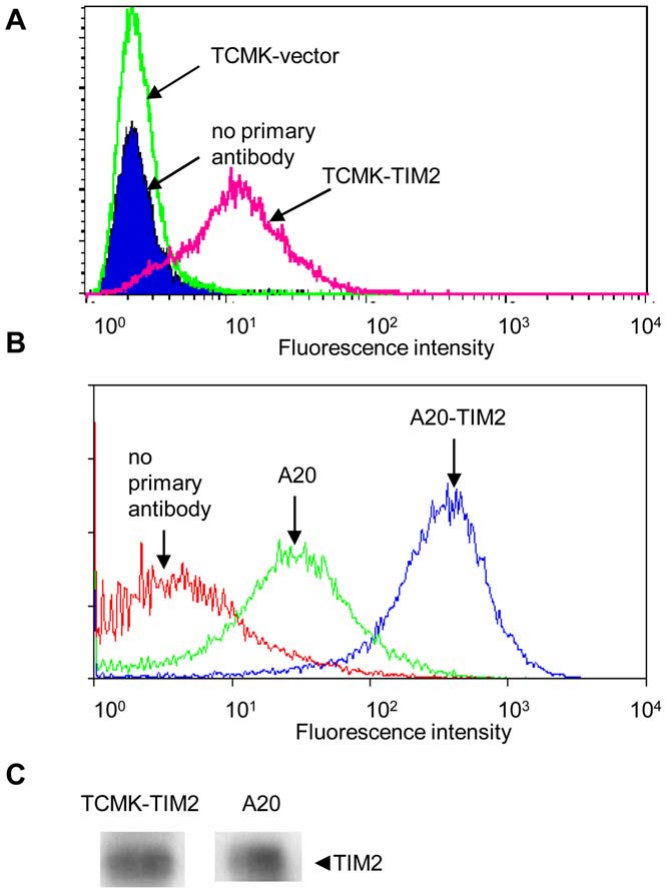
TIM-2 is present on the cell surface of transfected cells. A: Flow cytometry was performed on non-permeabilized TCMK TIM-2 transfectants and control vector transfectants. B: Flow cytometry was performed on A20 cells (mouse lymphocyte that expresses endogenous TIM-2) and A20 cells transfected with TIM-2 for over-expression. Cells were incubated with anti-TIM-2 monoclonal antibody (100 µg/ml), followed by FITC-linked anti-mouse secondary antibody (100 µg/ml). Primary antibody was omitted in controls. C: TIM-2 was immunoprecipitated from TIM-2 transfected TCMK cells and from A20 cells and analyzed by western blotting. Lanes were derived from the same blot exposed for the same length of time; intervening irrelevant lanes have been cropped out.

To confirm that TIM-2 was appropriately displayed on the cell surface of transfected cells, we used flow cytometry. We compared levels of cell surface TIM-2 in transfectants to A20 cells, a mouse lymphocyte cell line that expresses endogenous TIM-2, as well as to A20 cells that had been transfected with a TIM-2 expression vector to increase expression of TIM-2 [22]. As expected, A20 cells expressed endogenous TIM-2, and this increased in TIM-2 transfected A20 cells (Fig. 2). As shown in Figure 2, TIM-2 expression on the cell surface of TCMK-TIM-2 cells was significantly higher than in vector controls, and of the same order of magnitude as seen in A20 cells expressing endogenous TIM-2. Immunoprecipitation confirmed that levels of TIM-2 in untreated TCMK-TIM-2 transfectants were approximately equivalent to levels of endogenous TIM-2 in untreated A20 cells (Fig. 2C).

**Figure 3:**
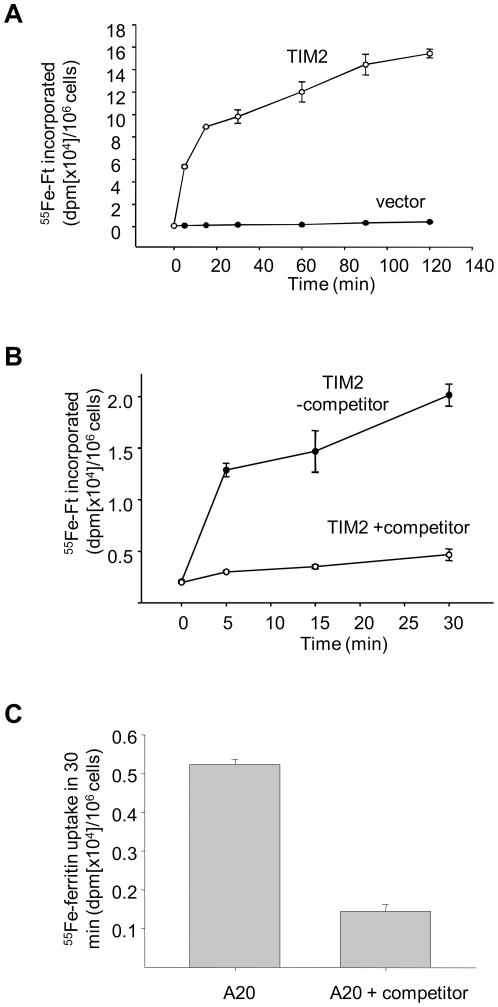
Specific uptake of HFt in TIM-2 expressing cells. A. HFt was labeled with^55^Fe and incubated at a final concentration of 4 nM at 37°C with TIM-2 positive TCMK cells or TCMK vector controls. B. TIM-2 TCMK cells were incubated with ^55^Fe-HFt in the presence or absence of 100-fold unlabeled iron-saturated HFt. C. A20 cells were incubated for 30 minutes with ^55^Fe-HFt in the presence or absence of 100-fold unlabeled HFt. Shown are means and standard deviations of triplicate determinations in a single experiment. The experiment was repeated 3 times with similar results.

To test whether cell surface TIM-2 could function as an uptake mechanism for ferritin-bound iron, recombinant HFt was labeled with ^55^Fe. TCMK-TIM-2 and vector controls were incubated with ^55^Fe-HFt at 37°C. Cells were collected at intervals, washed, and incorporated ^55^Fe was measured. As shown in Figure 3A, iron uptake increased in a time-dependent manner in TIM-2 transfectants. No uptake was seen in vector controls. Uptake in TIM-2 cells was specific, and could be effectively competed by a 100 fold molar excess of unlabeled ferritin (Fig. 3B). ^55^Fe-HFt was also taken up by A20 cells, and uptake was inhibited by ferritin (Fig. 3C).

### TIM-2 expression does not increase with iron chelation

Since iron transporters are frequently regulated by iron [36], we queried whether levels of TIM-2 were responsive to changes in levels of exogenous iron. We first examined the sequence of TIM-2 for the presence of a canonical iron responsive element (IRE). This element is present in the untranslated regions of mRNAs encoding proteins of iron uptake, storage, and export and serves as a target site for binding of IRPs. No canonical IRE-like sequence was evident in TIM-2 (data not shown).

**Figure 4:**
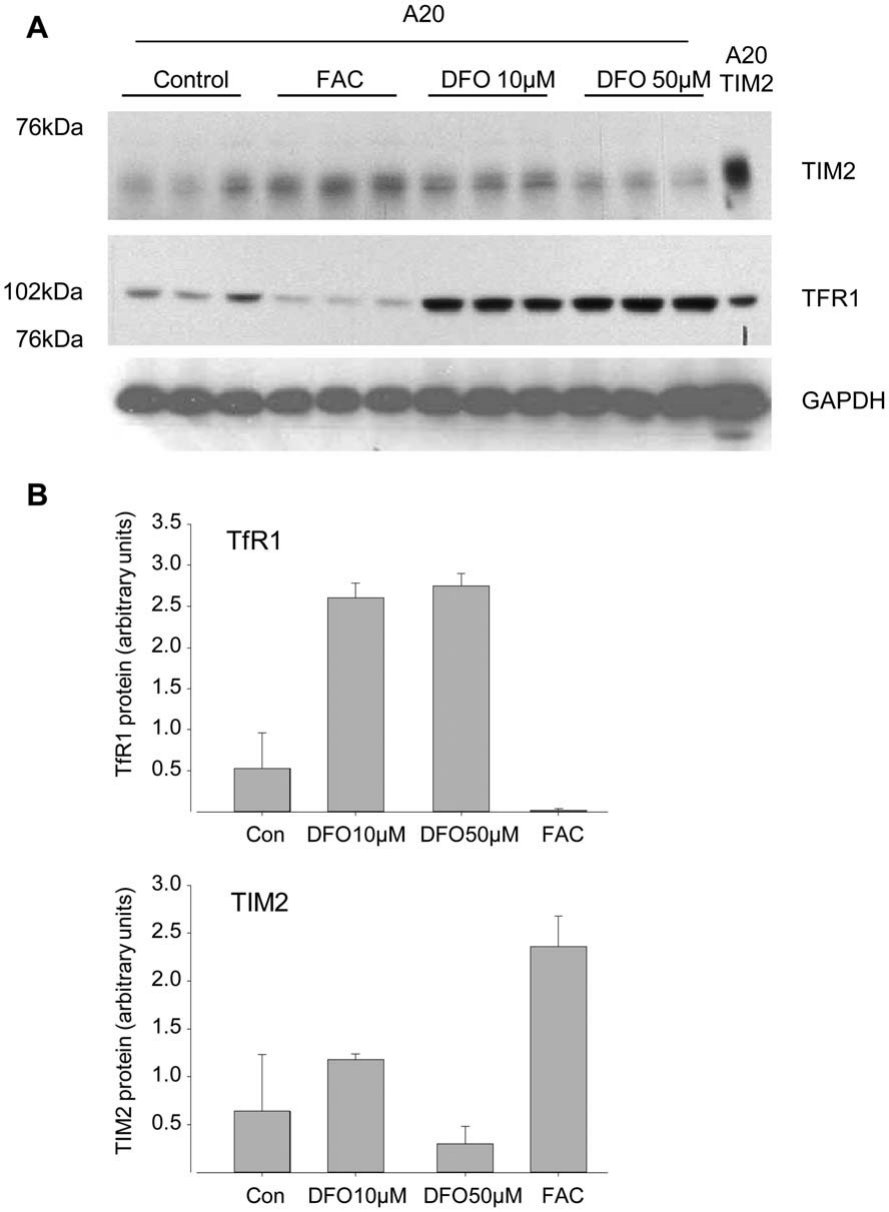
Endogenous TIM-2 is not regulated by iron chelation. Triplicate cultures of A20 cells were incubated with control medium or medium containing 1 mM ferric ammonium citrate (FAC), 10 µM deferoxiamine (DFO) or 50 µM deferoxiamine for 24 hrs. Cells were harvested and expression of TIM-2 and TfR1 analyzed by western blotting. GAPDH was used as a loading control. B. Image intensities of the triplicate determinations shown in (A) were quantified and normalized to GAPDH; means and standard deviations are shown. The experiment was repeated 3 times independently with similar results.

We then directly measured the effect of altered iron levels on expression of endogenous TIM-2 in A20 cells. Cells were either left untreated, or treated with 1 mM ferric ammonium citrate, 10 µM deferoxamine (DFO, an iron chelator) or 50 µM DFO for 24 h, and TIM-2 expression was assessed by western blotting. Expression of TfR1 was measured simultaneously as a control for effects of iron treatment. As shown in Fig. 4, treatment with iron decreased TfR1, whereas treatment with the iron chelator DFO increased TfR1, as expected. In contrast, there was no significant effect of DFO on TIM-2, and iron appeared to increase rather than decrease TIM-2. These results indicate that TIM-2 is not regulated by iron status in the same way as TfR1.

### Ferritin uptake by TIM-2 proceeds via an endocytic pathway

**Figure 5:**
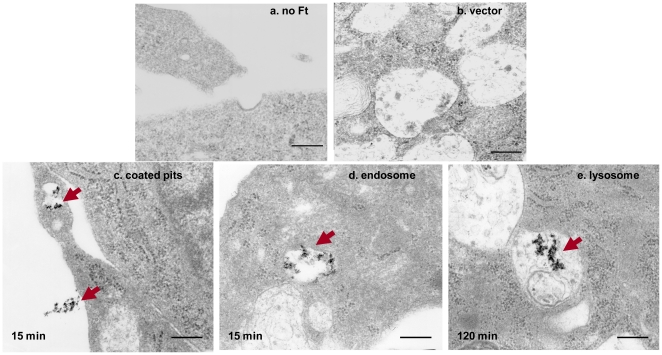
HFt bound to TIM-2 traffics to endosomes and lysosomes. A. TCMK-TIM-2 and vector controls were incubated with iron-loaded HFt for 15 and 120 minutes at 37°C and observed by electron microscopy. A. TCMK-TIM-2 cells without added HFt. B. Vector transfectants treated with iron-loaded HFt at 37°C for 120 minutes. C. HFt in early endosomes of TCMK-TIM-2 cells following 15 minutes of incubation with iron-loaded HFt at 37°C. D. HFt in endosomes or multivesicular bodies after 15 minutes incubation with TCMK-TIM-2 cells at 37°C. E. HFt in lysosomes after 120 minutes incubation with TCMK-TIM-2 cells at 37°C. Arrows indicate iron-loaded HFt. 55,000X magnification. Scale bar equals 0.36 µm.

**Figure 6:**
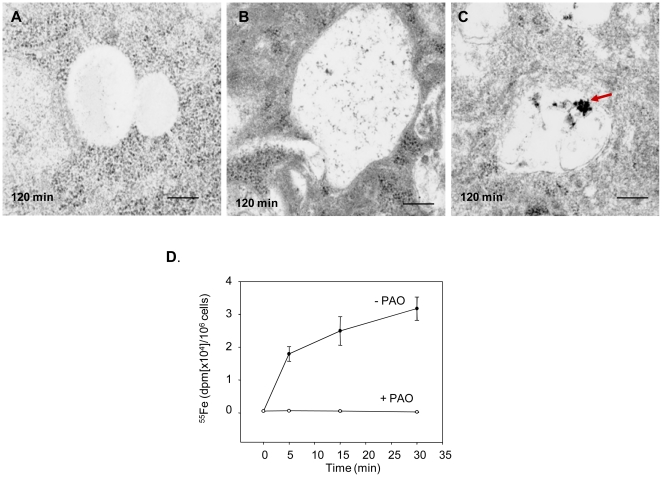
Uptake of HFt by TIM-2 is temperature dependent. A. TCMK TIM-2 cells were incubated without HFt at 37°C. B. TCMK-TIM-2 cells were prechilled and incubated on ice for 2 hours in the presence of HFt saturated with iron. C. TCMK-TIM-2 cells were incubated with iron-saturated HFt at 37°C for 2 hours. Scale bar equals 0.36 µm. D. TIM-2 transfectants were incubated with ^55^Fe-HFt in the presence or absence of of 10 µM phenylarsine oxide (PAO), an endocytosis inhibitor. Shown are means and standard deviations of triplicate determinations. The experiment was repeated 3 times independently with similar results.

To track the fate of iron-containing ferritin following uptake by TCMK TIM-2 cells, we used electron microscopy, which enables visualization of ferritin due to its electron-dense iron core. As shown in Figure 5, when TIM-2 cells were incubated with iron-saturated HFt for 15 minutes at 37°C, HFt was observed in coated pits and endosomes (Fig. 5C and 5D). At 120 minutes, ferritin appeared in lysosomes (Figure 5E). Uptake of ferritin was temperature-dependent, and was sharply reduced at 4°C (Fig. 6A–C). These observations suggested that ferritin uptake was mediated by an endocytic pathway. To confirm this result, we measured the uptake of ^55^Fe-HFt in the presence of the endocytosis inhibitor, phenylarsine oxide (PAO). As shown in Figure 6D, ferritin-mediated iron uptake was completely blocked in cells treated with 10 µM PAO.

### Intracellular iron released from HFt enters a bioavailable pool

**Figure 7:**
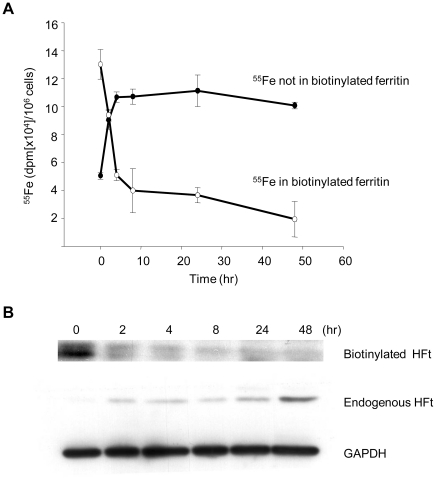
Iron enters a metabolically available pool following TIM-2-mediated uptake of HFt. TCMK TIM-2 transfectants were incubated with 4 nM biotinylated ^55^Fe-HFt at 37°C for 2 hrs. The cells were washed 3 times with PBS and then changed to normal growth medium. Cell lysates were prepared at 0, 2, 4, 8, 24, 48 hrs and biotin-^55^Fe-HFt was precipitated by incubation with streptavidin conjugated beads. Extracts depleted of exogenous biotinylated ferritin following incubation with streptavidin beads were used to assess endogenous HFt. **A**. ^55^Fe in biotinylated HFt and cellular fraction depleted of biotinylated HFt. Means and standard deviations of triplicate determinations are shown. **B**. Biotinylated HFt and endogenous ferritin were detected by western blotting. GAPDH was used as a loading control.

To trace the fate of HFt-bound iron in cells, we prepared biotinylated HFt, labeled it with ^55^Fe, and incubated it with TCMK-TIM-2 cells for two hours. Cells were then washed, and biotinylated HFt and its associated radioactivity were measured by pulldown using streptavidin beads. ^55^Fe in the cell lysate depleted of biotinylated HFt was also measured. As shown in Figure 7A, under these conditions iron was released from exogenous (biotinylated) ferritin and transferred to a cellular fraction. Release of iron occurred concomitantly with degradation of biotinylated ferritin, consistent with processing through the lysosome (Fig. 5). To test whether iron released from exogenous ferritin became biologically available, we measured levels of endogenous ferritin, since this protein is translationally upregulated by iron [1]. As shown in Figure 7B, release of iron from exogenously supplied (biotinylated) ferritin was paralleled by an increase in endogenous ferritin. The kinetics of the increase in endogenous ferritin were similar to those previously described following degradation of ferritin in the lysosome [37]. These results suggest that ^55^Fe released from HFt delivered through TIM-2 is able to enter a biologically available pool, increase levels of intracellular ferritin, and alter cellular iron status.

## Discussion

We previously demonstrated that TIM-2 serves as a specific receptor for HFt in mouse cells [22]. Results presented here demonstrate that iron bound to HFt can serve as a cellular iron source in mouse B cells or in mouse kidney cells that express TIM-2. Following uptake in TIM-2-positive cells, HFt traffics to endosomes and subsequently enters lysosomes (Fig. 5). This pathway differs from the classical pathway of iron delivery involving transferrin-mediated uptake of iron via TfR1, in which transferrin recycles to the cell surface following iron release in the endosome. Nevertheless, iron released from ferritin enters a metabolically available pool, and is able to upregulate synthesis of endogenous ferritin (Fig. 7).

Our work is consonant with other reports demonstrating both the ability of ferritin to enter the lysosome and to serve as an iron source. For example, recent work has shown that iron chelation can induce entry of cytosolic ferritin into lysosomes, where it undergoes degradation [38]. Treatment of cells with cationic ferritin similarly led to delivery of ferritin to lysosomes, iron release, and induction of cytosolic ferritin synthesis [37]. In human cells, TfR1 serves as a receptor for HFt [25], and may functionally substitute for TIM-2 [22]. Receptor-mediated uptake of HFt similarly directs ferritin to lysosomes in these cells [25]. Collectively, these results imply the existence of a mechanism for the shuttling of iron from the lysosome to the cytosol. Candidate transport mechanisms that may mediate iron efflux out of the lysosome have recently been identified [39], [40].

The relationship between TIM2 and Scara5, another recently identified ferritin receptor that functions in iron trafficking during development [24], is unclear. Unlike TIM-2, which selectively binds HFt, Scara5 binds LFt. Similar to our results with TIM-2, Scara5 mediates endocytosis of ferritin and ensuing delivery of iron to cells. It was suggested that Scara5 may functionally substitute for TfR1 in selected tissues of the kidney during development [24]. The relationship between expression of Scara5 and TIM-2 has not been examined. In the developing mouse, Scara5 is expressed in stromal and capsular cells of the kidney as well as in the airway, developing aorta, muscle bundles and gonadal epithelia [24]; in the adult mouse, Scara5 is expressed in epithelial cells of the testis, bladder, trachea, adrenal, skin, lung, and ovary [41]. This spatial distribution demonstrates little overlap with TIM-2, which is expressed in adult B cells, bile duct epithelial cells, renal tubules and oligodendrocytes, suggesting that in the adult, expression patterns of these ferritin receptors are largely independent. However, further work will be required to determine whether Scara5 and TIM-2 exhibit any temporal or functional relationship.

Whether the TIM-2 pathway contributes in a substantial way to iron import remains to be determined. Our results demonstrate that TIM-2 can mediate uptake of ferritin and its associated iron; however, the amount of iron delivered to cells through this pathway will depend on the iron content of the ferritin, which can vary over more than 2 orders of magnitude [42].

It is possible that the iron content of ferritin may influence the ultimate cellular effect of TIM-2. For example, ferritin exhibits immunosuppressive effects, inhibiting the proliferation of T and B cells and the differentiation of myeloid cells [9], [11], [43], [44]. Since lymphocytes express TIM-2, it has been suggested that this anti-proliferative effect of ferritin could be mediated through TIM-2 [44]. We speculate that if ferritin contains little or no iron, it may serve as a signaling molecule to induce such anti-proliferative or apoptotic [45] effects. Alternatively, if receptor-bound ferritin contains iron, it may serve as an iron source to support cell proliferation. Delivery of iron through ferritin has previously been proposed to occur in the developing kidney [24], and in macrophage-mediated delivery of ferritin-bound iron to erythroid precursors [16]. Our results indicate that TIM-2 has the potential to function as an iron delivery mechanism, but further work will be required to elucidate the circumstances under which that mechanism is activated.

Although specific binding of ferritin to cell surfaces has been repeatedly documented [21], [46], [47], [48], [49], [50], the source of ferritin that binds to TIM-2 and other ferritin receptors *in vivo* remains unknown. In our experiments, cells were exposed to 4 nM ferritin, which is within the range found in the serum of patients with iron overload and other inflammatory conditions such as Stills disease, hemophagocytic syndrome, etc. [51]. However, the majority of subunits in serum ferritin resemble ferritin L more closely than ferritin H [52], [53], and since TIM-2 preferentially binds HFt, it is not clear that serum ferritin represents a likely source of ferritin ligand for TIM-2. Nevertheless, ferritin is a 24 subunit protein, and most natural ferritins are heteropolymers of both H and L subunits. Since the number of H subunits required for effective binding of TIM-2 has not yet been determined, ferritin proteins that contain a preponderance of L subunits may nonetheless bind to TIM-2. In addition, small amounts of H subunit-rich ferritin are present in the serum and can increase in certain pathological conditions [53], [54]. An alternative potential source of ferritin is local secretion. For example, the macrophage, which can serve as a source of circulating ferritin [53] may also secrete ferritin locally [16], [55], and this may serve as a paracrine source of ferritin for uptake by TIM-2. Indeed HFt was identified as a soluble ligand secreted by macrophage cell lines during the cloning of TIM-2 [22]. HFt is also released by hepatocytes [45]. Future studies will be required to trace the physiological source of the HFt ligand.

Our results indicate that expression of TIM-2 is not increased by iron chelation (Fig. 4). This distinguishes TIM-2 from other iron uptake pathways. For example, TfR1, which mediates Tf-dependent uptake of iron, and DMT-1, which mediates transport of ferrous iron, are post-transcriptionally regulated by iron status [4]. For these transporters, regulation is mediated by IRE elements on the 3′ end of the mRNA. Our sequence inspection demonstrated no obvious candidate IRE elements in the sequence of TIM-2. However, since IRE elements may exhibit substantial divergence in primary sequence [56], we cannot rule out the presence of a non-canonical IRE element in TIM-2. The lack of response of TIM-2 to iron chelation that we observed is different from observations in oligodenodrocytes, in which expression of TIM-2 was reported to be iron-regulated [23]. Oligodendrocytes have no TfR1 to mediate iron uptake [23], may depend heavily on the TIM-2 pathway, and may have alternative mechanisms of regulation not found in other cell types. In contrast, in A20 cells, iron uptake is mediated by two pathways: the classical TfR1 pathway [57] and the TIM-2 pathway described here. It is possible that uncoupled regulation of TfR1 and TIM-2 may permit differential activation of these pathways. For example, in A20 cells (and other cells that express both TfR1 and TIM-2), TIM-2 may serve as a backup pathway that can respond to environmental changes in HFt regardless of cellular iron status.
